# Editorial: Machine learning for peptide structure, function, and design

**DOI:** 10.3389/fgene.2022.1007635

**Published:** 2022-09-20

**Authors:** Ruiquan Ge, Chuan Dong, Juexin Wang, Yanjie Wei

**Affiliations:** ^1^ School of Computer Science and Technology, Hangzhou Dianzi University, Hangzhou, China; ^2^ Hangzhou Institute of Advanced Technology, Hangzhou, China; ^3^ Key Laboratory of Combinatorial Biosynthesis and Drug Discovery, Ministry of Education, and School of Pharmaceutical Sciences, Wuhan University, Wuhan, China; ^4^ Department of BioHealth Informatics, Indiana University Purdue University Indianapolis, Indianapolis, IN, United States; ^5^ Shenzhen Institutes of Advanced Technology, Chinese Academy of Sciences, Shenzhen, China

**Keywords:** machine learning, functional peptides, deep learning, drug design, peptide therapeutics

Peptides with a length from 2 to 50 amino acids play important roles in the biological process and functions. Because of their wonderful variety of biological properties, peptide-based therapy has been a potential treatment for many diseases for decades. Meanwhile, peptide sequence, structure, and function are closely related, especially the relationship between structure and function. However, obtaining the structure or function of the peptides with wet experiments is costly, laborious, and time-consuming. In recent years, because of the obvious advantages of traditional machine learning and deep learning technology, these methods have been widely used in various protein or peptide structure and function predictions such as many kinds of site prediction, various interactions prediction, drug-targets prediction, and so on.

This Research Topic explores the new technologies and applications of machine learning on peptide structure and function prediction. We are pleased to see that the authors of the 12 accepted papers introduce the research progress and application of the latest machine learning techniques in peptide-related problems. They are related to peptide treatment of disease, inter-residue distance or contact, binding site prediction, drug targets, community-specific function landscape for peptides, and related discussions.

Peptide-based therapy has become a new potential method of disease treatment in recent decades years. Compared with traditional disease treatments, such as radiation therapy and chemotherapy, therapeutic peptides could avoid the obvious side effects of traditional disease treatments to guarantee precise treatment. Furthermore, most therapeutic peptides have the characteristics of high specificity, low production cost, low toxicity, easy synthesis, modification, etc. In this topic, three tools are proposed to predict therapeutic peptides, which are blood-brain barrier penetrating peptides (BBPpredict), antihypertensive peptides (Ensemble-AHTPpred), antiparasitic peptides (i2APP). Comparing with the experimental results of nine classifiers on the five-fold cross-validation and independent testing datasets, Chen et al. (BBPpredict) use a random forest method with optimal features selected by three feature scoring methods to predict the blood-brain barrier penetrating peptides (BBPs). In addition, they construct an online web service of BBPpredict to help researchers predict and find novel BBPs to accelerate the development of new drugs to treat central nervous system (CNS) diseases. Furthermore, Lertampaiporn et al. propose a robust ensemble machine learning model to identify antihypertensive peptides (Ensemble-AHTPpred). Ensemble-AHTPpred integrates various computed features and optimally weighted classifiers to improve the performance of the model. Moreover, i2APP proposed by Jiang et al. employs a two-step machine learning framework to identify antiparasitic peptides (APPs). It utilizes multi-feature extraction, feature selection with maximum information coefficient, and random down-sampling technology to improve the performance of models to identify APPs efficiently.

In addition, six papers pay attention to the inter-residue relationship, interaction, and binding sites. Zhang et al. propose DueDis to predict the inter-residue distance with duet deep learning models. DuetDis use the 1D and 2D complementary feature sets and high-quality multiple sequence alignment (MSA) to improve the prediction performance in the fused features. Peptide inter-residue contact maps determine its topological structure. Gu et al. utilize graph convolutional neural networks (GCN) and two different dimensional residual neural network architectures (1D ResNet and 2D ResNet) to capture global and local information, respectively. The compared experiments demonstrate its effectiveness on four different test datasets exceptionally on the long-range contact types. Furthermore, drug–target interactions (DTIs) are a hot topic in new drug discovery. Zheng et al. develop DTI-BERT to predict DTIs based on pre-trained Bidirectional Encoder Representations from Transformers (BERT) and deep learning methods. In the DTI-BERT model, sequence features are extracted by the pre-trained BERT for the proteins. And drug information is generated by Discrete Wavelet Transform (DWT) from drug molecular fingerprints. Then, a deep learning network is employed to judge the interaction using contrastive loss and cross-entropy loss in a few target families. In addition, Zhou et al. develop SSH2.0 to predict the hydrophobic interaction risk of monoclonal antibodies. SSH2.0 trains a new support vector machine-based ensemble model with the selected CKSAAGP features. Compared to the previous SSH, SSH2.0 performs better and may be a good web tool for researchers. In addition, protein post-translational modifications (PTMs) play crucial roles in diverse biological processes, affecting the protein’s function. Nowadays, various computational tools are developed to identify disease-associated PTM sites. In this issue, Indriani et al. propose ProtTrans-Glutar model to predict whether a protein sequence includes a glutarylation site. ProtTrans-Glutar extracts several kinds of feature sets such as the distribution feature, enhanced amino acid composition (EAAC), and ProtT5-XL-UniRef50, a pre-trained transformer-based model. Meanwhile, random under-sampling and XGBoost classifiers are used to train the model. Besides, Xu et al. propose AttnTAP to predict the binding of T cell receptor (TCR) and peptide with a dual-input deep learning framework to precisely predict the TCR-peptide binding. For AttnTAP, a bi-directional long short-term memory model (BiLSTM) model and attention mechanism with different weights for amino acids are employed to predict TCR-peptide binding effectively.

The remaining three articles analyze and discuss peptide-related problems from a relatively broad perspective. Vajjala et al. develop a metaBP toolkit to construct a community-specific function landscape for bacterial peptides from meta-genomic samples. The toolkit metaBP and metaBP-ML can discover and annotate bacterial peptides from a natural microbial community. It may give us a new research perspective to better understand the characteristics of bacterial peptides. For another research work, Liu et al. reveal and verify that traditional peptide quantitative structure-activity relationship (pQSAR) strategies only model the genome-wide domain–peptide interaction (DPI) qualitatively or semi-quantitatively because of disordered peptide conformation and potential interactions between peptide residues. For the last work, Wang et al. design a three-step pipeline to discover drug targets using cinnamon in cardiovascular diseases and metabolic syndrome. Through pathway filter, combined network construction, and biomarker prediction and validation to quantitative analysis of the effects of peptide-protein complexes as drug targets, 17 peptide-protein complexes are identified as the cinnamon targets in 6 peptides and 4 proteins. The pipeline based on network analyses using machine learning may foster new drug discovery based on peptides.

In conclusion, this special issue involves several hot topics in solving peptide-related problems using currently popular machine learning techniques. These efforts will help accelerate the development of vaccines and new drugs. Additionally, we hope these works can attract more researchers to focus on the related fields. Moreover, we thank all the reviewers and authors for their efforts and contributions to this special issue.

